# An efficient energy management scheme using rule-based swarm intelligence approach to support pulsed load via solar-powered battery-ultracapacitor hybrid energy system

**DOI:** 10.1038/s41598-024-53248-0

**Published:** 2024-02-17

**Authors:** Muhammad Shahid Wasim, Muhammad Amjad, Muhammad Abbas Abbasi, Abdul Rauf Bhatti, Akhtar Rasool, Abdur Raheem, Ahmed Ali, Baseem Khan

**Affiliations:** 1 Faculty of Engineering, The Islamia University of Bahawalpur, Bahawalpur, Punjab 63100 Pakistan; 2 Department of Electrical Engineering and Technology, Government College University, Faisalabad, Punjab 38000 Pakistan; 3 Department of Electrical Engineering, University of Botswana, UB 0061, Gaborone, Botswana; 4 Department of Electrical and Electronic Engineering Technology, Faculty of Engineering and the Built Environment, University of Johannesburg, Johannesburg, 2092 South Africa; 5 Hawassa University, Hawassa, Ethiopia

**Keywords:** Energy infrastructure, Energy storage, Renewable energy

## Abstract

This work presents an energy management scheme (EMS) based on a rule-based grasshopper optimization algorithm (RB-GOA) for a solar-powered battery-ultracapacitor hybrid system. The main objective is to efficiently meet pulsed load (PL) demands and extract maximum energy from the photovoltaic (PV) array. The proposed approach establishes a simple IF-THEN set of rules to define the search space, including PV, battery bank (BB), and ultracapacitor (UC) constraints. GOA then dynamically allocates power shares among PV, BB, and UC to meet PL demand based on these rules and search space. A comprehensive study is conducted to evaluate and compare the performance of the proposed technique with other well-known swarm intelligence techniques (SITs) such as the cuckoo search algorithm (CSA), gray wolf optimization (GWO), and salp swarm algorithm (SSA). Evaluation is carried out for various cases, including PV alone without any energy storage device, variable PV with a constant load, variable PV with PL cases, and PV with maximum power point tracking (MPPT). Comparative analysis shows that the proposed technique outperforms the other SITs in terms of reducing power surges caused by PV power or load transition, oscillation mitigation, and MPP tracking. Specifically, for the variable PV with constant load case, it reduces the power surge by 26%, 22%, and 8% compared to CSA, GWO, and SSA, respectively. It also mitigates oscillations twice as fast as CSA and GWO and more than three times as fast as SSA. Moreover, it reduces the power surge by 9 times compared to CSA and GWO and by 6 times compared to SSA in variable PV with the PL case. Furthermore, its MPP tracking speed is approximately 29% to 61% faster than its counterparts, regardless of weather conditions. The results demonstrate that the proposed EMS is superior to other SITs in keeping a stable output across PL demand, reducing power surges, and minimizing oscillations while maximizing the usage of PV energy.

## Introduction

Energy harvesting from the sun is becoming more popular than other renewable energy resources due to abundant solar insolation, low operating and maintenance costs, etc.^1^. However, its high response time and output fluctuations due to ecological factors such as solar irradiance, temperature, clouds, dust, and partial shading conditions (PSCs) make it difficult to run instantaneous pulsed loads (PL) such as high-power radars, electromagnetic launch and recovery systems, ships, and electric vehicles (EVs)^2^. Integrating photovoltaic (PV) energy with high-energy-density batteries and high-power-density ultracapacitors (UCs) addresses the issue of high response times and output fluctuations. However, an energy management scheme (EMS) is necessary to control the energy flow among PV, battery bank (BB), UCs, and PL to achieve the unique benefits of each component in a solar-powered BB-UC hybrid system^3,4^.

Different EMS and associated control techniques have been investigated for various applications in the literature^5–18^. A decentralized control system is proposed, which utilizes droop control to distribute power between the BB and UCs^19^. It suffers from poor current sharing and voltage drops in the DC grid due to droop action. Another EMS is proposed based on a full current type polynomial control approach for BB-UC hybrid system^20^. Its complexity and high computational intensity limit its real-time applicability and increase hardware requirements. A BB-UC hybrid energy storage system (HESS) designed to enhance battery life while addressing PL challenges is introduced in^21^. It demonstrates improvements in battery life compared to individual batteries and UC systems. However, the charging scheme for any energy storage device (ESD) is not discussed as it assumes pre-charged ESDs. A decentralized EMS using fuzzy logic control (FLC) has been presented for ship power systems that successfully distributes power between ESDs^13^. This work considers low-frequency PL for a short duration, which requires further investigation. Moreover, fuzzification is a complex process and requires additional memory for lookup tables. An EMS based on a linear quadratic regulator (LQR) is proposed for the BB-UC hybrid system^8^. It offers control advantages, but with increased computational complexity and sensitivity to model errors. It needs precise parameter adjustments in dynamic and uncertain operating conditions. A composite model predictive control (MPC)-based decentralized dynamic power sharing strategy for HESS is presented^22^. Although it has its merits, it also has limitations. These include the need for real-time model updates and constraints on prediction horizons, which can make it less suitable for highly dynamic and complex systems. The system incorporates a low-pass filter (LPF) to divide power sharing between ESDs^7^. It uses a proportional-integral (PI) controller to stabilize the DC bus voltage and reduce battery usage by integrating UCs. Using a linear PI controller with LPF can introduce inherent lag characteristics that negatively impact current regulation. A solar-powered BB-UC HESS for PL management is proposed^9^. It employs a rate limiter to distribute power between ESDs and a sliding mode controller (SMC) to regulate current flow to the battery. Various heuristic methods like particle swarm optimization (PSO) and genetic algorithms (GA) have been explored for power management in solar-powered BB-UC HESS^18^. An EMS for a solar-powered BB-UC HESS is proposed using the minimum principle of Pontryagin^23^. It is limited to deterministic problems and restricted to a single type of load, with no comparison to high-frequency PL.

In summary, EMS can be classified into three main categories: classical techniques^7–10^, intelligent control methods^11–16^, and metaheuristic optimization approaches^2,18,24–26^. Classical techniques such as deadbeat control, droop control, sliding control, and filtration-based control require a precise system model to function efficiently^20^. Furthermore, these are sensitive to variations in model parameters^11^. Artificial intelligence (AI) techniques such as fuzzy logic, neural networks, and machine learning are reliable for slow-dynamic applications while handling uncertainties in future load patterns^12,27^. The primary flaw in AI techniques is the development of their rules, which requires expert knowledge and experience. Hundreds of trained data sets are used for accurate prediction, which takes time to train^23,28^. Another drawback is the significant memory demand to save customized values. Optimization-based strategies try to minimize or maximize the objective function. MPC can minimize the effects of variation in model parameters while maintaining performance^17,22,25,29,30^. However, its effectiveness depends on its precise modeling and sophisticated computational setup^2,23^. This setup requires a lot of effort in design and constraint-sensitivity analysis. Metaheuristic optimization techniques such as PSO, water wave optimization, GA, and white shark optimization (WSO) are used in various EMS^18,24,31^. Although PSO has gained more popularity than others, it suffers from slow and premature convergence^32^. Additionally, it has a tendency to converge to local optima and may require a large number of iterations to obtain good solutions, leading to high computational costs. Thus, its limitations suggest the need for alternative metaheuristic techniques that can address these issues and provide more efficient and robust solutions.

The control techniques previously used are inadequate as they do not take into account one or more of the following factors: PL demand, charging UC through BB, MPPT under PSCs, and ensuring system stability under different operating conditions. This article aims to highlight the benefits of using a solar-powered BB-UC hybrid energy system to address the issues mentioned above in a single manuscript. For this, an EMS based on a rule-based grasshopper optimization algorithm (RB-GOA) is presented, focusing on two key aspects of interest. The first aspect of EMS is to support PL demands and ensure UC charging by BB. This is managed by creating two independent management layers: a long-term energy management layer (EML) and a short-term power management layer (PML). EML narrows the search space by considering the operating limits of the components. It activates various operational modes with the help of IF-THEN rules based on different scenarios. PML uses a GOA to establish optimal real-time power sharing among PV, BB, and UC within the search space defined by EML. RB-GOA optimizes the objective function and produces reference signals to control two bidirectional converters (BDCs) that are connected between the BB and the DC link, as well as between the UC and the DC link. The second aspect is to develop an MPPT scheme under PSC to take advantage of the maximum available PV energy. This is achieved through the implementation of a GOA that controls the duty ratio of a boost converter connected to the common DC link.

A detailed analysis is carried out to assess and compare the performance of the proposed technique with other well-known swarm intelligence techniques (SITs) such as the cuckoo search algorithm (CSA), gray wolf optimization (GWO), and salp swarm algorithm (SSA). Evaluation is performed for a number of different scenarios, including PV alone without the use of an energy storage device (ESD), PV with maximum power point tracking (MPPT), variable PV with constant load, and variable PV with PL. The comparative analysis demonstrates that the proposed approach performs better than the other SITs in terms of power surge reduction during load transition, oscillation mitigation, and MPPT speed. In particular, it reduces power surge in the variable PV constant load case by 8% to 26% as compared to other SITs. Additionally, it reduces oscillations more than two to three times as quickly as the compared algorithms. Furthermore, in the variable PV with PL case, it minimizes the power surge by 6 to 9 times. Additionally, its MPP tracking speed is roughly 29% to 61% faster than other SITs compared under uniform irradiance and shaded conditions. These results demonstrate the superiority of the proposed technique over the other SITs in maintaining constant output across PL demand and optimizing the use of PV energy.

The proposed work addresses critical challenges in a solar-powered BB-UC hybrid system, specifically high response times, output fluctuations, and the efficient integration of PV energy with ESDs. The research introduces an EMS based on RB-GOA to optimize real-time power share between PV system, BB, and UCs. The EMS includes both long-term energy management and short-term power management to adapt to the dynamic nature of PV systems. Furthermore, an optimized MPPT scheme is presented to tackle variation of solar irradiance to maximize the energy harvesting from the PV array. Comparative analysis of RB-GOA with other SITs highlights its superior performance in terms of power surge reduction, oscillation mitigation, and MPPT speed. The proposed system contributes in providing the improved and reliable performance of solar-powered BB-UC HESS under dynamic real-world conditions, which can significantly impact the field of PV energy and energy management.

The rest of the work is divided into the following sections: The modeling of components of the hybrid system is presented in “Modeling of system components” section. “Problem formulation” section presents the formulation of a control problem with an objective function.  “Set of rules” section outlined the set of rules. GOA is explained in “Grasshopper optimization algorithm” section. The combination of the rule-based control and GOA is presented in “Rule-based GOA” section and parameters selection of the SITs used is given in “Parameters selection of SITs” section. Simulation results for various case studies are presented in “Results and discussions” section. Finally, the conclusion is presented in “Conclusion” section.

## Modeling of system components

**Figure 1:**
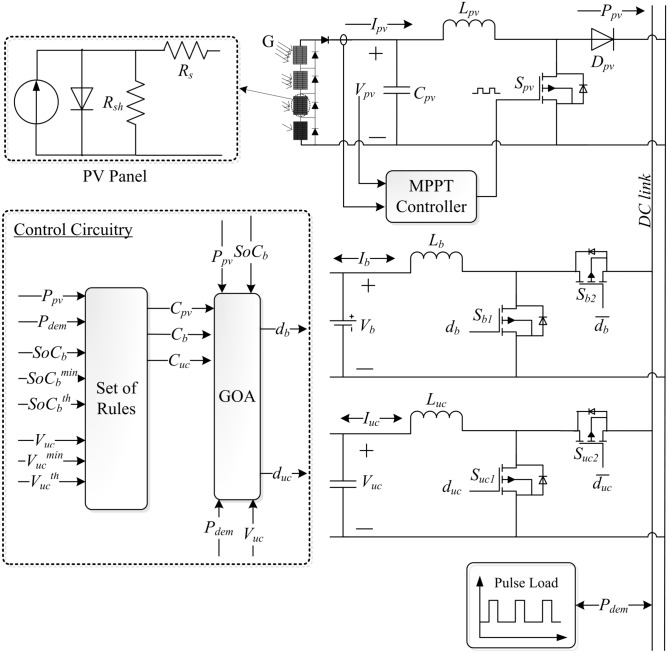
Proposed EMS circuitry.

Figure 1 shows the proposed system in which a boost converter is used to link the PV system to the DC link. The energy storage devices (ESDs) comprise a combination of BB and UC, both of which are connected to the DC link through their respective bidirectional converters. The three sources collaborate to power the PL. PV energy serves as the primary source; the battery is used to compensate for a power shortage or store surplus power; and the UC is used to compensate for instantaneous load variation. The MPPT technique is used to extract the maximum power from PV via the boost converter. A rule-based algorithm controls each bidirectional converter, managed by an energy management system (EMS).

### PV system modeling

A PV system is composed of a PV array, a boost converter, and a GOA-based MPPT algorithm, as shown in Fig. 1. The main objective of this PV system is to extract the maximum available energy from the PV array, irrespective of the ecological conditions. The output current $(I_{pv})$ Eq. (1) of a PV module is obtained by its single-diode model^6,18,24^. The main external factors that affect $I_{pv}$ are incident irradiance (*G*) and temperature (*T*). In addition, internal factors that can significantly reduce its output are series resistance $(R_s)$ and shunt resistance $(R_{sh})$. $R_s$ is resistance to current flow, which is due to the ohmic contact between the metal and the internal resistance of the semiconductor, and $R_{sh}$ shows a leakage current that depends on the quality of the module surface^32^.

1 $$\begin{aligned} \begin{aligned} I_{pv}&= N_p I_{sc} \left( 1+\alpha _{sc} \left( T-T_r\right) \right) \frac{G}{G_r} \\&\quad - N_p I_{sr} \left( \frac{T}{T_r}\right) ^3 \left( e^\frac{qE_G \left( \frac{1}{T_r}- \frac{1}{T}\right) }{nk} \right) \left( e^\frac{q\left( \frac{V_{pv}}{N_s}+\frac{(I_{pv} R_s)}{N_p} \right) }{nkT}-1\right) \\&\quad -\left( \frac{\frac{N_p}{N_s} V_{pv}+I_{pv} R_s }{R_{sh}}\right) \end{aligned} \end{aligned}$$

$I_{sc}$: short circuit current of the modules with its temperature coefficient $\alpha _{sc}$, $T_r$, $G_r$: reference temperature and solar insolation respectively, $I_{sr}$, *n*: reference saturation current and non-ideality factor of the diode, respectively, $E_G$: band gap energy of the cell material, $V_{pv}$: module voltage, *k*: Boltzmann’s constant, *q*: charge on an electron, $N_p$: the number of parallel solar cells, and $N_s$: the number of cells in series. The power of a single PV module ($P_{mod}$) is given in Eq. (2)^2^.

2 $$\begin{aligned} P_{mod}= max\left( I_{pv} \times V_{pv}\right) \end{aligned}$$

The output power of the PV array $(P_{pv})$ given in Eq. (3) is obtained by connecting the PV modules $(N_{pv})$ in various combinations.

3 $$\begin{aligned} P_{pv}= \sum _{k=1}^{N_{pv}} P_{mod} ^ {(k)} \end{aligned}$$

### Battery bank modeling

BB is a high-energy density device that is used as a backup power source to run the load when PV energy is not available^5^. It reduces the negative effects of the intermittent behavior of PV power by storing surplus energy. A bidirectional converter ($BDC_b$) is used to link a BB to a common DC link in the circuit model depicted in Fig. 1. A macroscopic modeling method is sufficient for BB sizing since the goal is not to examine the internal chemistry of the BB. Therefore, for this work, an equivalent electrical model of the battery based on the Rint structure is used. It consists of an internal resistance connected in series with a variable open-circuit voltage that depends on the state of charge ($SoC_b$) and temperature^23^. $SoC_b$ states the remaining stored energy in the BB. Therefore, it is considered a crucial metric in the analysis of BB under various operational circumstances. It is updated using the Coulomb counting (CC) approach with the following formula in Eq. (4)^33,34^.

4 $$\begin{aligned} SoC_b(t)=SoC_b^0-\frac{1}{Q_b}\int _{0}^{t} I_b(t) dt \end{aligned}$$

where $SoC_b^0$ is the initial $SoC_b$, $Q_b$ is the capacity of the BB, and $I_b$ is the current taken or supplied through the BB. The sign of $I_b$ is positive when the BB is supplying power to the load and negative when the BB is being charged. In this work, the lithium-ion battery block model available in MATLAB/Simulink is used. The minimum power needed $(Req\_BB\_Pwr)$ by BB to raise $SoC_b(t)$ to an upper limit ($SoC_b^u$) in the time step $\Delta t$ with $N_{b}$, the number of batteries is given by Eq. (5)^35^.

5 $$\begin{aligned} Req\_BB\_Pwr = \frac{\left( SoC_b^{u}-SoC_b(t)\right) \times Q_{b} \times N_{b} }{\Delta t} \end{aligned}$$

Similarly, the remaining power of the battery (Eq. 6) is the maximum power that can be continuously delivered for the time step $\Delta t$ before $SoC_b(t)$ approaches its lower limit $SoC_b^l$.

6 $$\begin{aligned} Avl\_BB\_Pwr = \frac{\left( SoC_b(t)-SoC_b^l\right) \times Q_{b} \times N_{b} }{\Delta t} \end{aligned}$$

### Ultracapacitor modeling

Electric double-layer capacitors (EDLCs), UCs, or SCs are energy storage capacitors. Both batteries and UCs are electrochemical devices, but no electrochemical reaction occurs in the energy storage mechanism of UCs. UCs store energy electrostatically by polarizing an electrolytic solution. This dissimilar operating principle of UCs makes its features very different from the battery. UCs possess a considerably high power density, low energy density, high charge-discharge efficiency, and more than a million cycles of life^21^. They can operate efficiently from − 40 °C to + 70 °C. Energy storage in UCs has a direct relation with the square of their terminal voltage $(V_{uc})$ and can be calculated using Eq. (7).

7 $$\begin{aligned} E_{uc}= & {} \frac{1}{2} C_{uc} \left( V_{uci}^2-V_{ucf}^2 \right) =P_{uc} t \end{aligned}$$

8 $$\begin{aligned} C_{uc}= & {} 2P_{uc} t\frac{1}{\left( V_{uci}^2-V_{ucf}^2 \right) } ) \end{aligned}$$

where $E_{uc}$ is the stored energy in Joules, $C_{uc}$ is the capacitance in Farads, $V_{uci}$ is the initial voltage, and $V_{ucf}$ is the final voltage across the UC terminals in volts, $P_{uc}$ is the power in watts and *t* is the discharge time in seconds. Energy can be obtained between the rated volts and zero volts, so 100% stored energy can be hypothetically extracted but 5% energy is dissipated in heat^20^. However, a designer keeps a protection boundary to avoid the reverse charge of unstable cells.

## Problem formulation

The main objective is to meet the pulsed load demand ($P_{dem}$) within the specified limits and to get maximum PV energy under all weather conditions. To achieve the objective, a multilevel EMS is presented, focusing on two key aspects of interest. The first aspect is to support PL demands and ensure UC charging through BB. This is managed by creating two independent management layers: a long-term energy management layer (EML) and a short-term power management layer (PML). EML narrows the search space with the help of a set of IF-THEN rules. PML produces reference signals with the help of GOA to control $BDC_i$ using these rules and the search space defined by EML. The search space contains the operating limits of the components. The central equation to fulfill $P_{dem}(t)$ at any instant *t* is given in Eq. (9).

9 $$\begin{aligned} P_{dem}(t)= \sum C_i(t)\times P_i(t), \hspace{0.5cm} i~\in ~\left( PV,BB, UC\right) , \hspace{0.5cm} C_i(t)~\in ~[-1,1] \hspace{0.25cm} \forall ~t \end{aligned}$$

Here, $P_i(t)$ is the power supplied by the $i_{th}$ component and $C_i(t)$ is the capacity assignment factor for better power management between components^36^. $C_i(t)$ is restricted by the limits of the upper bound $UB_i$ and the lower bound $LB_i$ i.e., $C_{i}(t) \in [LB_i, UB_i]$ where $LBi \ge -1$ and $UB_i \le 1$. The $UB_i$ defines the discharging ability and the $LB_i$ describes the charging ability of the component. Typical values of the bounds are $LB_i \in [-1,0]$ for charging and $UB_i \in [0,1]$ for discharging. If an ESD is being charged, the power sign is shown as negative, and if energy is supplied, the sign is positive. Moreover, it is assumed that the PV system always supplies the generated energy so, $C_{pv}(t)$ $\in$ [0, 1] without the negative component.

The life of the BB depends on its dis(charge) rate ($C-rate$), number of cycles, and depth of discharge ($DoD_b$), $DoD_b = 1 - SoC_b$. Moreover, the discharge of pulse power from the battery-alone system significantly reduces its life^21^. Therefore, BB life can be improved by controlling its $C-rate$, $SoC_b$, and discharging it without pulse current. For this, a set of constraints in Eq. (10) on $P_{b}(t)$ is applied defining the minimum and maximum power supplied or taken through the BB. Secondly, $SoC_b$ is restricted within the specified upper $SoC_b^u$ and lower $SoC_b^l$ values given in Table 3. Furthermore, the pulse load that is greater than the BB discharging limit ($P_{dem} > P_b^{dlim}$) is directed to the UC.

10 $$\begin{aligned} {\left\{ \begin{array}{ll} P_i^{clim} \le P_i(t) \le P_i^{dlim} \\ P_i^{clim} \le 0 \le P_i^{dlim} \hspace{01cm} i~\in ~\left( BB, UC\right) , \hspace{0.5cm} \forall ~t \\ \end{array}\right. } \end{aligned}$$

**Figure 2:**
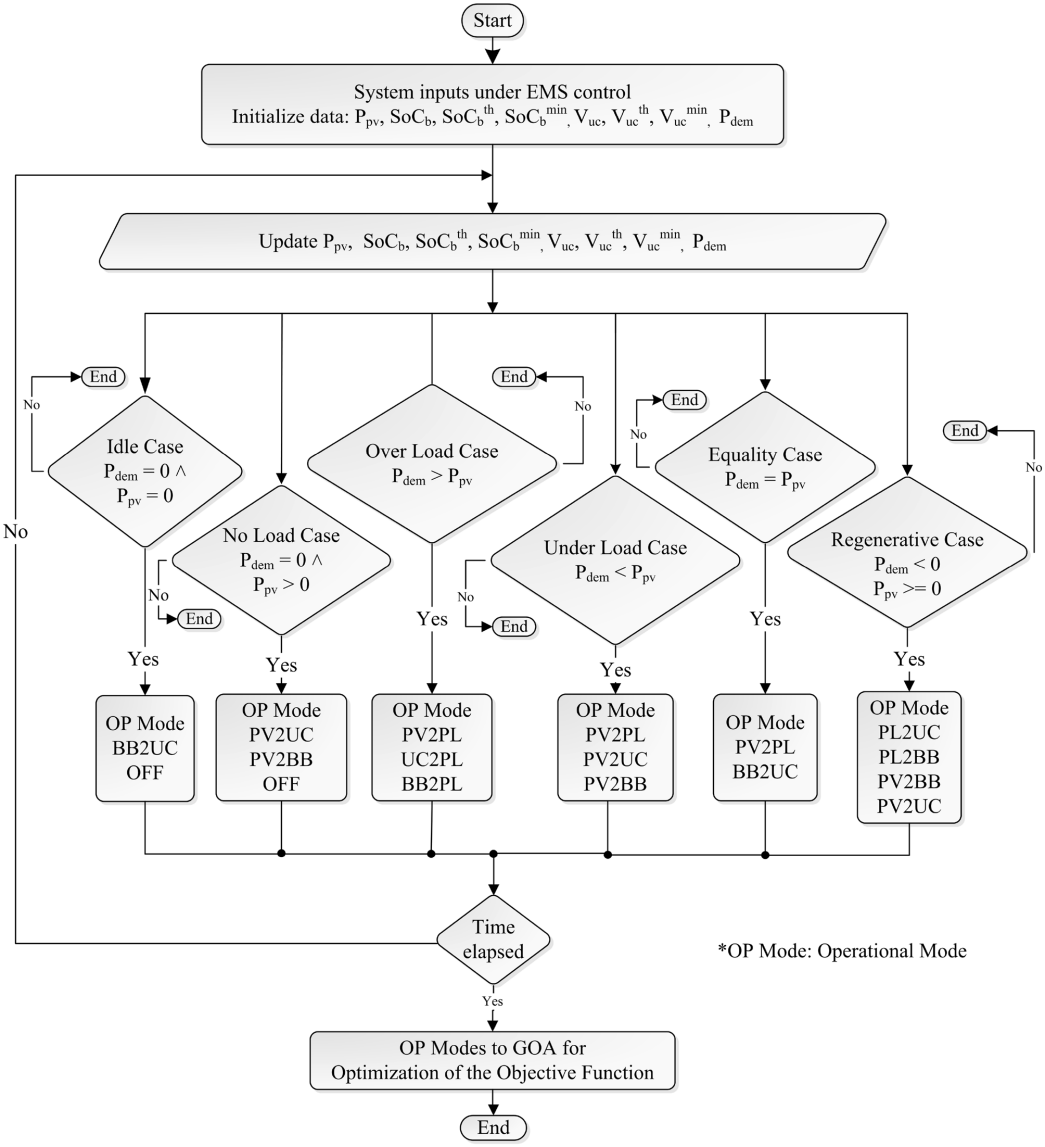
Operation control of proposed EMS.

Here, $P_i^{clim}$ is the maximum charging limit of the components and $P_i^{dlim}$ is the maximum power that can be supplied to the load by the components. The PV system does not consume energy, so $P_{pv}^{clim} = 0$. Moreover, it always supplies the maximum energy available, so $P_{pv}^{dlim} = 1$. As mentioned above, UC energy storage can be controlled by its terminal voltage ($V_{uc}$), and battery life can be improved by managing $SoC_b$. Therefore, using $V_{uc}$, $SOC_b$, $P_{dem}$, and $P_{pv}$ with their limits in Eq. (10), the EML is defined by a set of IF-THEN rules. Table 1 represents the determined rules and Fig. 2 represents the flow chart of the defined rules.

The solution search space defined by EML is restricted to $C_{i}(t) \in [LB_i, UB_i]$, so PML decisions must be implemented within the search space, taking into account the load and available energy. PML defines online power allocations to components by controlling $BDC_i$ with the help of GOA using the search space. This is necessary to supply power to the PL without interruptions and deteriorating the component’s performance. Therefore, the objective function is to minimize the difference between $P_{dem}$ and $P_i$ in the time interval *j* as in Eq. (11).

11 $$\begin{aligned} ObjF[j] = \min _{C_{pv},C_b,C_{uc}} \Biggl |P_{dem}[j] - \Bigl [C_{pv}[j] \times P_{pv}[j] + C_b[j] \times P_b[j] + C_{uc}[j]\times P_{uc}[j]\Bigr ]\Biggr | \end{aligned}$$

GOA is used to minimize the objective function. It produces the duty ratio signals $d_b$ and $d_{uc}$ to control $BDC_{b}$ and $BDC_{uc}$, respectively. If $d_b$ is positive, then $S_b1$ ($1^{st}$ MOSFET in $BDC_b$) is ON and the BB supplies energy to the load. If $d_b$ is negative (${\bar{d}}_b$), then $S_b2$ ($2^{nd}$ MOSFET in $BDC_b$) is ON and the BB receives the charging current from the system. Similar is the case for $d_{uc}$. The objective function can be rewritten as Eq. (12)

12 $$\begin{aligned} ObjF[j] = \min _{d_b,d_{uc}} \Biggl |P_{dem}[j] - \Bigl [P_{pv}[j] + d_b[j] \times P_b[j] + d_{uc}[j] \times P_{uc}[j]\Bigr ]\Biggr | \end{aligned}$$

The power $P_b (j)$ and $P_{uc}(j)$ can be calculated using Eqs. (13) and (14) in which $V_b^{oc}, V_{uc}^{oc}$ are the open circuit voltages and $\delta _b, \delta _{uc}$ are the SoC gains of BB and UC, respectively, given in Table 3.

13 $$\begin{aligned} P_b(j)= & {} \left( V_b^{oc}(j-1)+\delta _b \cdot SoC_b(j-1)\right) \cdot I_b^{ref} \end{aligned}$$

14 $$\begin{aligned} P_{uc} (j)= & {} \left( V_{uc}^{oc}(j-1)+\delta _{uc} \cdot SoC_{uc}(j-1) \right) \cdot I_{uc}^{ref} \end{aligned}$$

The second aspect is to track the MPP of the PV system under uniform and partially shaded conditions. GOA is used to generate a controlled duty ratio *d* of the boost converter using voltage and current as inputs. The power generated $(P_{pv})$ for a specific duty cycle $d_i$ using $k_{th}$ iteration and $i_{th}$ swarm number is defined as Eq. (15).

15 $$\begin{aligned} P_{pv}(d_{i}^{(k)}) > P_{pv}(d_{i}^{(k-1)} ) \end{aligned}$$

where $P_{pv}$ is the power received for a specific $d_i$ at iteration number *k* for the $i_{th}$ GH in GOA. Here, $d_i$ is the position of $i_{th}$ GH that is to be optimized. The algorithm aims to find the optimal *d* that maximizes the power received by the system. At each iteration, the power received for a specific $d_{i}^{(l)}$ is evaluated. This power value represents the fitness or objective value of the GH. The algorithm compares this power value in the current iteration *k* with the power value in the previous iteration $k-1$. The inequality in Eq. (15) serves as a criterion to determine whether the GHs have improved their positions (*d*) in terms of maximizing the power received. If the power received in the current iteration is greater than the power received in the previous iteration, this implies that the GHs have made progress and have moved toward an optimal *d*. Repetition of this process over several iterations is key to moving GHs towards the optimal *d* and thus reaching the maximum output power target.

## Set of rules

The EML foundation of the proposed EMS is a rule-based strategy using simple IF-THEN operators. The IF statement chooses various operational scenarios, and the THEN statement executes the modes. The scenario is the situation under which the operational mode is activated. The operational mode is the mechanism that decides where the actual energy is coming from or where it is going to the different system’s components.

IF-THEN rules play a crucial role in defining the search space and guiding the optimization process. These rules are typically based on a set of conditions (IF part) and corresponding actions (THEN part). Specific examples and case scenarios are given below.

- *Example 1*: IF - the $SOC_b$ is below 20%. THEN - start charging the battery using the available energy source. This rule ensures that the battery is always charged sufficiently to meet the power demands of the load.
- *Example 2*: IF - the pulse load demand is high. THEN - increase the power discharge from the UCs. This rule ensures a steady power supply to meet peak pulse loads. These are just a few examples of how IF-THEN rules can be used to define the search space for an optimization algorithm.
- *Impact of the IF-THEN rules on the optimization process:* Reduces the search space to improve the convergence speed of the optimization algorithm and eliminates infeasible solutions to improve the quality of the final solution. Bias the optimization algorithm towards specific regions of the search space to achieve specific objectives. For instance, in Example 1, the algorithm is biased towards solutions that maintain the $SOC_b$ above 20%. This can be useful for applications where a long battery life is essential. In Example 2, the algorithm is biased towards solutions that use the UCs to meet the peak demands of the pulse load.

**Table 1:** Set of rules.

Case	IF condition	THEN	Mode
Idle Case:	$V_{uc} < V_{uc}^{th}$ & $SoC_b > SoC_b^{th}$	$C_b \in [0,1]$	BB2UC
		$$C_{uc} \in [-1,0]$$	
		$$C_{pv} \in [0, 0]$$	
$P_{dem}(t)= 0 \, \& \, P_{pv}(t) = 0$	$SoC_b < SoC_b^{th}$	$C_b \in [0,0]$	OFF
		$$C_{uc} \in [0,0]$$	
		$$C_{pv} \in [0,0]$$	
No Load Case:	$V_{uc}< V_{uc}^{max}\, \& \,P_{pv}<P_{uc}^{clim}$	$C_b \in [0,0]$	PV2UC
		$$C_{uc} \in [-1,0]$$	
		$$C_{pv} \in [0, 1]$$	
	$V_{uc}< V_{uc}^{max}\, \& \,P_{pv}>P_{uc}^{clim}\, \& \,SoC_b < SoC_b^{max}$	$C_b \in [-1,0]$	PV2UC, PV2BB
		$$C_{uc} \in [-1,0]$$	
		$$C_{pv} \in [0,1]$$	
$P_{dem}(t)= 0\, \& \,P_{pv}(t) > 0$	$V_{uc} = V_{uc}^{max}\, \& \,SoC_b < SoC_b^{max}$	$C_b \in [-1,0]$	PV2BB
		$$C_{uc} \in [0,0]$$	
		$$C_{pv} \in [0,1]$$	
	$V_{uc} = V_{uc}^{max}\, \& \, SoC_b = SoC_b^{max}$	$C_b \in [0,0]$	OFF
		$$C_{uc} \in [0,0]$$	
		$$C_{pv} \in [0,0]$$	
Over Load Case:	$OLC< P_b^{dlim}\, \& \, SoC_b>SoC_b^{min}$	$C_b \in [0,1]$	PV2PL, BB2PL
		$$C_{uc} \in [0,0]$$	
		$$C_{pv} \in [0, 1]$$	
	$OLC < P_b^{dlim} \, \& \, SoC_b=SoC_b^{min} \, \& \, V_{uc} > V_{uc}^{min}$	$C_b \in [0,0]$	PV2PL, UC2PL
		$$C_{uc} \in [0,1]$$	
		$$C_{pv} \in [0,1]$$	
$P_{dem}(t) - P_{pv}(t) =OLC > 0$	$OLC> P_b^{dlim} \, \& \, V_{uc}> V_{uc}^{min} \, \& \, SoC_b>SoC_b^{min}$	$C_b \in [0,1]$	PV2PL, BB2PL, UC2PL
		$$C_{uc} \in [0,1]$$	
		$$C_{pv} \in [0,1]$$	
	$OLC> P_b^{dlim}\, \& \, V_{uc} > V_{uc}^{min}\, \& \, SoC_b = SoC_b^{min}$	$C_b \in [0,0]$	PV2PL, UC2PL
		$$C_{uc} \in [0,1]$$	
		$$C_{pv} \in [0,1]$$	
	$OLC >or< P_b^{dlim}\, \& \, V_{uc} = V_{uc}^{min}\, \& \, SoC_b = SoC_b^{min}$	$C_b \in [0,0]$	PV2UC
		$$C_{uc} \in [-1,0]$$	
		$$C_{pv} \in [0,1]$$	
Under Load Case:	$V_{uc} < V_{uc}^{max}$	$C_b \in [0,0]$	PV2PL, PV2UC
		$$C_{uc} \in [-1,0]$$	
		$$C_{pv} \in [0, 1]$$	
$P_{dem}(t) - P_{pv}(t) < 0$	$SoC_b < SoC_b^{max}$	$C_b \in [-1,0]$	PV2PL, PV2BB
		$$C_{uc} \in [0,0]$$	
		$$C_{pv} \in [0,1]$$	
Equality Case:	$V_{uc} < V_{uc}^{th}\, \& \, SoC_b > SoC_b^{th}$	$C_b \in [0,1]$	PV2PL, BB2UC
		$$C_{uc} \in [-1,0]$$	
		$$C_{pv} \in [0, 1]$$	
$P_{dem}(t) - P_{pv}(t) = 0$	$SoC_b < SoC_b^{th}$	$C_b \in [0,0]$	PV2PL
		$$C_{uc} \in [0,0]$$	
		$$C_{pv} \in [0,1]$$	
Regenerative Case:	$V_{uc} < V_{uc}^{max} \, \& \, \left( |P_{dem}|+P_{pv}>0\right) > P_{uc}^{clim}$	$C_b \in [-1,0]$	PL2UC, PV2UC, PL2BB
		$$C_{uc} \in [-1,0]$$	
		$$C_{pv} \in [0, 1]$$	
$P_{dem}(t) < 0,\, P_{pv}(t) \ge 0$	$V_{uc} = V_{uc}^{max} \, \& \, SoC_b < SoC_b^{max} \, \& \, P_{pv}>0$	$C_b \in [-1,0]$	PL2BB, PV2BB
		$$C_{uc} \in [0,0]$$	
		$$C_{pv} \in [0,1]$$	
Minimum values of system	$SoC_b = SoC_b^{min} \, \& \, V_{uc} = V_{uc}^{min} \, \& \, P_{pv}(t) = 0$	$C_b \in [0,0]$	OFF
		$$C_{uc} \in [0,0]$$	
		$$C_{pv} \in [0, 0]$$	

### PV2PL mode

PV2PL mode is used for energy transfer from a PV system to PL. This procedure is carried out using a boost converter with MPPT abilities, as demonstrated in Fig. 1. PV energy is consumed as a priority to power the load and to reduce the stress on the ESDs. The energy supplied $PV2PL\_E$ varies in the following situations.

Situation 1: If $P_{pv}$ is sufficient to handle the $P_{dem}$, then $PV2PL\_E$ is equal to the energy required by the load equation (16).

16 $$\begin{aligned} PV2PL\_E = P_{dem} \times \Delta t \end{aligned}$$

Situation 2: If the generated $P_{pv}$ is greater than $P_{dem}$, then $P_{pv}$ is equal to the energy supplied $P_{dem}$ plus the surplus energy that is used to charge the UC (mode PV2UC mode) and / or BB (mode PV2BB mode). The $PV2PL\_E$ in this situation is given below by Eq. (17).

17 $$\begin{aligned} PV2PL\_E = P_{dem} \times \Delta t \end{aligned}$$

Situation 3: If generated $P_{pv}$ is not enough to power the $P_{dem}$ alone, then the entire generated $P_{pv}$ and deficit amount of energy supplied by the UCs (mode UC2PL mode) and/or BB (mode BB2PL mode) is used to compensate $P_{dem}$. The PV energy obtained in this case is given in Eq. (18).

18 $$\begin{aligned} PV2PL\_E = P_{pv} \times \Delta t \end{aligned}$$

### PV2UC mode

Energy is transferred from PV to UCs abbreviated as PV2UC. When $P_{pv}$ is greater than $P_{dem}$ and the $V_{uc}$ is less than the upper voltage level $V_{uc}^{max}$, then this mode is activated. $P_{pv}$ utilization to charge the UCs is $2^{nd}$ priority after supplying $P_{dem}$ as explained in mode PV2PL. The energy supplied by PV to UC $(PV2UC\_E)$ can be calculated with the situations given below.

Situation 1: When $P_{pv} > P_{dem}$, then the surplus energy transferred to UC charging is the power required by the UC (Req_UC_Pwr) in time $\Delta t$ given by Eq. (19). The Req_UC_Pwr cannot exceed the $P_{uc}^{clim}$.

19 $$\begin{aligned} PV2UC\_E = Req\_UC\_Pwr \times \Delta t \end{aligned}$$

Situation 2: In the no-load condition $(P_{dem}=0)$ the available $P_{pv}$ is used to charge the ESDs. First, UC is given priority to be charged, and second, BB gets surplus energy through the mode PV2BB mode. The PV energy to charge UCs in this situation is given in Eq. (20).

20 $$\begin{aligned} PV2UC\_E = Req\_UC\_Pwr \times \Delta t \end{aligned}$$

Situation 3: In the regenerative case (mode PL2ESD mode), if the charging energy required by the UCs is greater than the regenerated energy, then the remaining energy to charge the UC is taken through $P_{pv}$. The $PV2UC\_E$ in Eq. (21)is the difference between the required UCs energy $(Req\_UC\_Pwr)$ to the regenerative energy $(Reg\_Pwr)$ in time $\Delta t$.

21 $$\begin{aligned} PV2UC\_E = \left( Req\_UC\_Pwr - Reg\_Pwr\right) \times \Delta t \end{aligned}$$

### PV2BB mode

In this mode, the BB gets charging energy from a PVS abbreviated as PV2BB. This mode is activated when the $P_{pv}$ is more than both the $P_{dem}$ and the energy required to charge the UC with the necessary condition $SoC_b < SoC_b^{max}$. The priority of PV energy is to run the load, the second priority is to charge the UCs and the third priority is to provide energy to BB. The energy supplied by PV to BB $(PV2BB\_E)$ can be calculated using different situations.

Situation 1: When generated $P_{pv}$ is more than $P_{dem}$ and $Req\_UC\_Pwr$ then the remaining energy is used to charge the BB in the system. The PV energy transferred to this situation is specified in Eq. (22).

22 $$\begin{aligned} PV2BB\_E = \left( P_{pv} - P_{dem} - Req\_UC\_Pwr\right) \times \Delta t \end{aligned}$$

Situation 2: In the no-load condition $(P_{dem}=0)$ the generated $P_{pv}$ is used to charge the UCs by means of mode PV2UC mode and the residual energy is used to charge the BB which is stated in Eq. (23).

23 $$\begin{aligned} PV2BB\_E = \left( P_{pv} - Req\_UC\_Pwr \right) \times \Delta t \end{aligned}$$

Situation 3: In regenerative situations (mode PL2ESD mode), in addition to regenerated energy, the available $P_{pv}$ is also utilized to charge the BB. PV energy in this situation is defined below in Eq. (24).

24 $$\begin{aligned} PV2BB\_E = \left( P_{pv} - Req\_UC\_Pwr \right) \times \Delta t \end{aligned}$$

### UC2PL mode

This mode allocates energy from UCs to PL, abbreviated as UC2PL. The minimum condition to trigger the mode is $V_{uc} > V_{uc}^{min}$, that is, the terminal voltage of the UCs should be higher than their lowest cutoff voltage. The energy supplied by the UCs to PL $(UC2PL\_E)$ varies under different conditions.

Situation 1: When the difference between the $P_{dem}$ and $P_{pv}$ is greater than the upper BB discharge limit ($P_b^{dlim}$) then the remaining energy is supplied by the UC to the PL. The energy transferred to the PL by the UC is given in Eq. (25).

25 $$\begin{aligned} UC2PL\_E = \left( P_{dem} - \left( P_{pv} + P_b^{dlim}\right) \right) \times \Delta t \end{aligned}$$

Situation 2: When $P_{pv} =0$ and $SoC_b = SoC_b^{min}$ then the energy supplied by the UC to the PL is as Eq. (26).

26 $$\begin{aligned} UC2PL\_E = P_{dem} \times \Delta t \end{aligned}$$

### BB2PL mode

The BB is the main ESD to supply the power to the load abbreviated as BB2PL. This is valid when $SoC_b > SOC_b^{min}$, i.e. the state of charge of the BB is higher than the cutoff $SoC_b^{min}$. The energy supplied by BB to PL $(BB2PL\_E)$ varies under different conditions.

Situation 1: When the $P_{pv}$ is not enough to run the load alone and ($P_{dem} - P_{pv} < P_b^{dlim}$), then the BB provides the energy deficiency to the loads computed as Eq. (27).

27 $$\begin{aligned} BB2PL\_E = \left( P_{dem} - P_{pv} \right) \times \Delta t \end{aligned}$$

Situation 2: When the condition ($P_{dem} - P_{pv} > P_b^{dlim}$) is satisfied then UCs help to provide the power to the PL and some of the energy is supplied by the BB to the PL as in Eq. (28).

28 $$\begin{aligned} BB2PL\_E = P_b^{dlim} \times \Delta t \end{aligned}$$

### BB2UC mode

Most published research work has not considered UC charging through the BB in HESS. The UC charging is done by an external energy-providing source such as PV energy, wind energy, and gas turbines, etc. To handle pulses in the load current, it is necessary to ensure the availability of UC energy. Therefore, this mode (BB2UC) is employed here to charge UCs through BB within the system so that the UC energy is mostly available to drive the PL. The initiation of this mode begins when the UC voltage is less than its threshold level $V_{uc} < V_{uc}^{th}$, i.e., $V_{uc}$ is dropped from the allowable threshold voltage level. The energy supplied by BB to UCs $(BB2UC\_E)$ varies under different conditions. Moreover, the required UC power (Req_UC_Pwr) cannot exceed $P_{uc}^{clim}$.

Situation 1: In the idle case where there is no $P_{dem}$ and no $P_{pv}$ the UCs get the following charging energy through the BB if $SoC_b > SOC_b^{th}$.

29 $$\begin{aligned} BB2UC\_E = Req\_UC\_Pwr \times \Delta t \end{aligned}$$

Situation 2: If the generated $P_{pv}$ is enough to power the $P_{dem}$ alone, then the UCs get the following charging energy (Eq. 30) through the BB.

30 $$\begin{aligned} BB2UC\_E = Req\_UC\_Pwr \times \Delta t \end{aligned}$$

Situation 3: When the difference between the $P_{dem}$ and $P_{pv}$ is less than $P_b^{dlim}$, $V_{uc} < V_{uc}^{th}$ and $SoC_b > SoC_b^{th}$ then the UCs are charged by the following energy Eq. (31).

31 $$\begin{aligned} BB2UC\_E = Req\_UC\_Pwr \times \Delta t \end{aligned}$$

### PL2ESD mode

The proposed system can also work for EVs. During the deceleration of the EVs ($P_{dem} < 0$), the energy is supplied back to ESD. Therefore, to store this regenerated energy $(Reg\_Enr)$ in the ESD, this PL2ESD mode is included. At first priority, the UCs take the $Reg\_Enr$ when $V_{uc} < V_{uc}^{max}$. The energy supplied by PL to ESDs varies under different conditions.

Situation 1: In the regenerative case where $P_{dem} > P_{uc}^{clim}$, the energy is transferred to UCs by Eq. (32).

32 $$\begin{aligned} PL2UC\_E = P_{uc}^{clim} \times \Delta t \end{aligned}$$

Situation 2: In the regenerative case where $P_{dem} < P_{uc}^{clim}$, the energy is transferred to UCs by Eq. (33).

33 $$\begin{aligned} PL2UC\_E = Req\_UC\_Pwr \times \Delta t \end{aligned}$$

Situation 3: In the regenerative case where $V_{uc} = V_{uc}^{max}$ or $Reg\_Pwr > P_{uc}^{clim}$ then BB is charged by $Reg\_Enr$ given in Eq. (34).

34 $$\begin{aligned} PL2BB\_E = Req\_BB\_Pwr \times \Delta t \end{aligned}$$

### OFF mode

There is a complete shutdown of the system when

Situation 1: In the idle case ($P_{dem} = P_{pv} =0$) where $V_{uc}> V_{uc}^{th}$ then the system goes to the OFF position.

Situation 2: In the case without load ($P_{dem} =0$ and $P_{pv} > 0$) when $V_{uc} = V_{uc}^{max}$ and $SoC_b = SoC_b^{max}$ then the system goes to the OFF position.

Situation 3: The system goes to the OFF position when $P_{pv} = 0$, $V_{uc} = V_{uc}^{min}$, and $SoC_b = SoC_b^{min}$.

**Figure 3:**
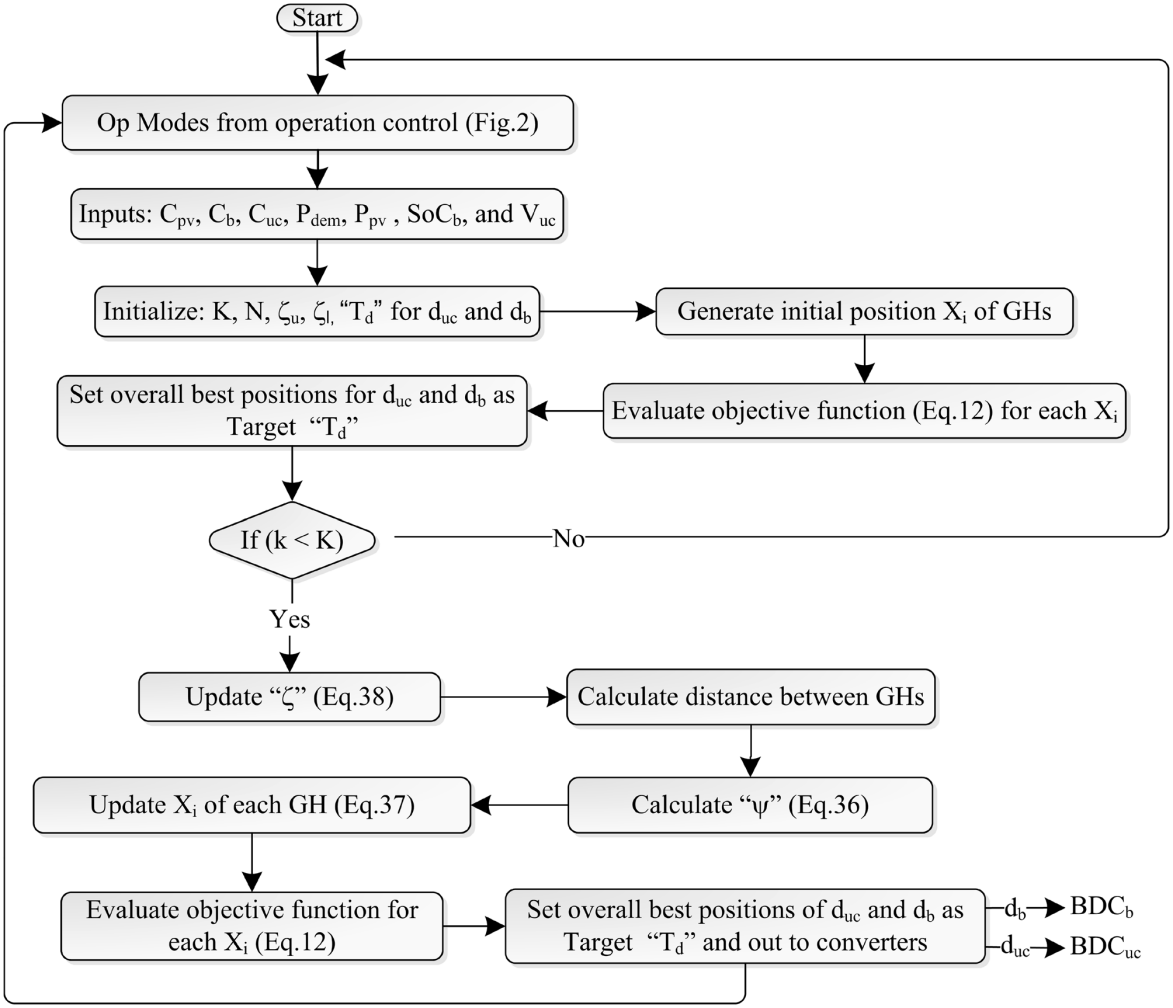
Flow chart of GOA.

## Grasshopper optimization algorithm

GOA is a recently developed population-based swarm intelligence method that simulates the social interaction and food search behavior of grasshoppers (GHs)^37^. They go through two stages in life; nymph and adult. The nymphs move slowly since they lack wings and consume all the plants in their path. Adult GHs have wings that form a swarm in the air that can quickly move to a wide area. Swarm activity in both nymphs and adults is what makes it special. Slow movement in small steps in the nymph stage is known as exploitation. The movement of large distances in the adult stage in a larger search space is known as exploration. The region where exploitation and exploration are equal is called the comfort zone. The position $\chi _i$ of $i_{th}$ GH is given by Eq. (35)^38,39^.

35 $$\begin{aligned} \chi _{i}= \sum \limits _{j=1,j\ne i}^{N} \psi (\left\| \xi _{j}-\xi _{i}\right\| ) \frac{\xi _{j}-\xi _{i}}{d_{ij}} -g{\hat{e}}_{g}+u{\hat{e}}_{w} \end{aligned}$$

*g* is the gravitational constant with its unit vector ${\hat{e}}_{g}$, *u* is the drift constant caused by the wind with its unit vector ${\hat{e}}_{w}$, $d_{ij}$ is the distance between the $i^{th}$ and $j^{th}$ GH, *N* is the total GHs. $\psi$ represents the attraction and repulsion forces between the GHs given below Eq. (36)^40^.

36 $$\begin{aligned} \psi (\gamma )= \alpha e^{(\frac{-\gamma }{\beta })}-e^{-\gamma } \end{aligned}$$

The terms $\alpha$ and $\beta$ stand for the length scale of attraction and the intensity of the attraction, respectively, at distance $\gamma$. The previous Eq. (35) is written as Eq. (37) to stop the GHs from immediately entering their comfort zone^41^.

37 $$\begin{aligned} \chi _{i}= \zeta \sum \limits _{j=1,j\ne i}^{N} \zeta \left( \frac{\nu _{u}-\nu _{l}}{2} \psi (\left\| \xi _{j}-\xi _{i}\right\| ) \frac{\xi _{j}-\xi _{i}}{d_{ij}} \right) +{\hat{T}}_{d} \end{aligned}$$

$\nu _{u}$, $\nu _{l}$ are the upper and lower limits of GH, ${\hat{T}}_{d}$ is the target value in dimension $d_{th}$. The parameter $\zeta$ is a decreasing coefficient, responsible for controlling the comfort zone and keeping a balance between exploration and exploitation.

38 $$\begin{aligned} \zeta = \zeta _u -k \frac{\zeta _u-\zeta _l}{K} \end{aligned}$$

$\zeta _{u}$, $\zeta _{l}$ are the maximum and minimum values, *k* is the current iteration, and *K* is the maximum number of iterations. Figure 3 shows the flow chart of the dynamic power shared by the GOA. At first, the GOA takes inputs from the operational modes defined by rule-based control as shown in Fig. 2, that is, $C_{pv}$, $C_{b}$, $C_{uc}$. It takes the other inputs such as $P_{dem}$, $P_{pv}$, $SoC_b$, $V_{uc}$ and initializes all the variables. The GOA randomly generates the initial position of each GH which is a potential solution to the optimization problem. It evaluates the objective function in Eq. (12) and calculates the initial output values related to all GH and assigns the best fit values to $d_b$ and $d_{uc}$ as target positions. The algorithm completes the iterative cycle using the equations shown in the Fig. 3. The optimized target positions values ($d_b$ and $d_{uc}$) are used to drive their respective converters as shown in Fig. 1.

## Rule-based GOA

A rule-based control (RBC) system utilizes a set of rules expressed in a logical format, such as the IF-THEN statements, to make decisions about how to control a given system. The RBC then uses these rules to determine the appropriate control actions based on the current state of the system. RBC is commonly used in complex systems where developing a mathematical model is difficult or impossible. Moreover, it is well-suited for applications where the control rules need to be transparent and easy to understand. Combining RBC and GOA for the solar-powered BB-UC hybrid energy system driving pulsed load has several benefits.

- *Exploration and exploitation:* The combination of RBC and GOA allows a balance between exploration and exploitation. The RBC defines guidelines for exploitation, while GOA explores the parameter space to improve the control strategy.
- *Versatility and adaptation:* RBC and GOA can adapt to different solar and load conditions through appropriate rules and an effective response system, respectively.
- *Global optimization:* The GOA enables the HESS to find the best operational points by considering variations in solar irradiance and pulsed loads, maximizing the utilization of PV energy.
- *Deterministic and robust behavior:* The use of RBC ensures that control actions are determined and unaffected by changes in system dynamics and external disturbances. Meanwhile, GOA provides an extra layer of robustness by optimizing the rules’ parameters and dealing with uncertainties. When combined, these techniques ensure reliable and consistent management of pulsed loads using a solar-powered HESS.
- *Flexibility:* RBC are flexible and easily adaptable to new situations and requirements. GOA can further improve flexibility by adapting to different operating conditions.

Overall, combining the advantages of RBC and GOA is a comprehensive approach to controlling and optimizing solar-powered BB-UC HESS with pulsed loads. It can help to address the challenges posed by pulsed loads while maximizing the utilization of PV energy and ensuring reliable performance.

The RB-GOA has several advantages over other SITs due to its inherent features, which include:

- *Robustness to noise:* RB-GOA utilizes GOA, which maintains robustness even when some solutions are influenced by noise.
- *Ability to learn complex relationships:* RB-GOA effectively captures complex and non-linear relationships between input and output variables.
- *Scalability to high dimensions:* RB-GOA uses a divide and conquer approach to handle high-dimensional data sets. It breaks down the problem into subproblems of lower dimensions, thereby maintaining performance even in high-dimensional spaces.

RB-GOA’s inherent features and design choices make it a promising approach for various applications.

## Parameters selection of SITs

The effective optimization of any technique often depends on the careful selection of its parameters. These parameter values and equations are sourced from established literature for each algorithm to ensure the robustness and reliability of the optimization approach. Table 2 provides the details about the parameter values of SITs with their equation and the source. For the CSA, these are obtained from^42^. They are essential for guiding the algorithm in its search for optimal solutions. Similarly, the SSA relies on parameters and equations borrowed from^43^, ensuring operation with well-defined settings. Furthermore, randomness is added through the variable $c_2$, which helps to explore the solution space more effectively. GWO takes its parameters and equations form^44^ which shows that the GOW linearly decreases $\alpha$ from 2 to 0.5 over time. Such adjustments help to improve the performance of the algorithm during the optimization process. In summary, this research is based on a thorough understanding of algorithms and their associated parameters, ensuring that the optimization approach is well-informed and capable of producing accurate and reliable results.

Moreover, in the experimental setup, the MATLAB version R2021a was used for data analysis and simulations. The computations were performed on a laptop featuring an Intel Core i5-6300U CPU, clocked at 2.40GHz with four processing cores, operating at approximately 2.5GHz. The laptop was equipped with 8192MB of RAM, providing the necessary computational resources to run the MATLAB simulations and ensure efficient data analysis.

**Table 2:** Parameters and equations of the algorithms^32^.

Algorithm	Equations	Values
CSA^42^	$d_k^{i+1} = d_k^i + \alpha \cdot \frac{\left| u \right| }{v^\frac{1}{\beta } } \cdot (d_{best} - d_i^k)$	$\alpha$ = 0.8
	$u \approx \mathcal {N}^v (0, \sigma _u^2)$	$\beta$ = 1.5
	$v \approx \mathcal {N}(0, \sigma _v^2)$	$\mathcal {N}$ = 4
	$$\sigma _u =\Bigg (\frac{\Gamma \left( 1 + \beta \right) \cdot \sin \left( \pi \cdot \frac{\beta }{2} \right) }{\Gamma \left( \frac{1 + \beta }{2} \right) \cdot \beta \cdot 2^{\left( \frac{\beta -1}{2} \right) }} \Bigg )$$	
	$$\sigma _v = 1$$	
GWO^44^	$\textbf{D} = \left| \textbf{C} \cdot \textbf{X}_{p}(t) - \textbf{X}(t) \right|$	
	$\textbf{X} (t+1) = \textbf{X}_p (t) - \textbf{A} \cdot \textbf{D}$	$a_u$ = 2
	$a = a_{l} - (a_{l} - a_{u} ) \cdot \tan \left( \frac{1}{\varepsilon } \cdot \frac{m}{M}\pi \right)$	$a_l$ = 0.5
	$A = 2a \cdot \text {random} - a$	$\epsilon$ = 4
	$C=2 \cdot \text {random}$	*M* = 120
SSA^43^	$$x_1^{(k)} = {\left\{ \begin{array}{ll} F^{(k)}+ c_1 \left( \left( U_b^{(k)}- L_b^{(k)}\right) c_2 + L_b^{(k)} \right) , &{} c_3 \ge 0.5 \\ F^{(k)}- c_1 \left( \left( U_b^{(k)}- L_b^{(k)}\right) c_2 + L_b^{(k)} \right) , &{} c_3 < 0.5 \end{array}\right. }$$	$U_b$ = 0.8
	$c_1 = 2 \cdot e^-\left( {\frac{4m}{M}}\right) ^2$	$L_b$ = 0.1
	$x_j^{(k)} = \frac{1}{2} \left( x_j^{(k)} + x_{j-1}^{(k)} \right)$	*M* = 120
		$c_2$ = random
GOA	Equation (37)	$\zeta _u$ = 1
	Equation (36)	$\zeta _l$ = 0.00004
	Equation (38)	$\alpha$ = 0.5 and $\beta$ = 1.5

## Results and discussions

A simple model in Fig. 1 is taken to assess the viability of the proposed algorithm. It consists of a PV array with MPPT capability using a boost converter, a BB with its $BDC_b$ and UC bank with its $BDC_{uc}$. The PV array generates $I_{pv}$ and $V_{pv}$ upon receiving the irradiance from the visible light spectrum. The boost converter increases $V_{pv}$ to $V_{dclink}$. The ripple current required by MOSFET’s ON/OFF is provided by $C_{pv}$ inserted between the PV array and the converter. GOA is used to track the MPP of the system by monitoring $I_{pv}$ and $V_{pv}$. Six Sun-Power modules (SPR-315E-WHT-D) are utilized to provide a maximum $P_{pv}$ of 1.89 kW in the system. A shaded and an unshaded PV array condition is taken to evaluate PV performance. The EML is controlled by a simple RB technique in which the inputs specified in Table 3 are $P_{pv}$, $P_{dem}$, $SoC_b$, $SoC_b^{min}$, $SoC_b^{th}$, $V_{uc}$, $V_{uc}^{min}$ and $V_{uc}^{th}$. Here, $SoC_b^{min}$ is the minimum allowable $SoC_b$ after which the system cannot take the power from BB and $SoC_b^{th}$ is the threshold $SoC_b$ after which it cannot charge the UC but can drive the load. Similarly, $V_{uc}^{min}$ is the minimum terminal voltage of the UC after which it cannot supply power to the load and $V_{uc}^{th}$ is the voltage level after which it can take power from the BB. The RB method performs the actions given in  “Set of rules” section and Table 1. It generates the output $C_{pv}$, $C_b$, and $C_{uc}$ that are fed to PML that uses GOA to optimize the objective function in Eq. (12). The system is assessed for PV alone without ESD, PV with MPP tracking, constant load variable PV, and varying load variable PV cases.

**Table 3:** Energy resources characteristics.

PV specifications		BB specifications		UC specifications	
Parameter	Value (Units)	Parameter	Value (units)	Parameter	Value (units)
$P_{mod}$	315 (W)	$Q_b$	7.5 (A h)	$Q_{uc}$	3.7 (A h)
$N_{pv}$	6	$N_b$	8	$N_{uc}$	2
[$P_{pv}^{min}$, $P_{pv}^{max}$]	[0, 1.89] (kW)	[$P_b^{clim}$, $P_b^{dlim}$]	[-0.6, 1] (kW)	[$P_{uc}^{clim}$, $P_{uc}^{dlim}$]	[− 1.2, 5] (kW)
[$V_{pv}^{oc}$, $V_{pv}^{mp}$ ]	[64.6, 54.7] (V)	[$SoC_b^{min}$, $SoC_b^{max}$]	[0.3, 1]	$[V_{uc}^{min},V_{uc}^{max}]$	[51, 102] (V)
[ $I_{pv}^{sc}$, $I_{pv}^{mp}$ ]	[6.14, 5.74] (A)	$SoC_b^{th}$	0.5	[$V_{uc}^{th}$, $V_{uc}^{oc}$]	[75, 102] (V)
$P_{pv}$ tolerance	± 5%	$V_b^{oc}$	96 (V)	$C_{uc}$	165 (F)
*T* coefficient of $P_{pv}$	− 0.38%/K	$\delta _b$	17.3 (V/−)	$\delta _{uc}$	80 (V/−)
*T* coefficient of $V_{pv}$	− 0.18%/K	$I_b^{ref}$	40 (A)	$I_{uc}^{ref}$	1200 (A)

### PV alone without ESD

**Figure 4:**
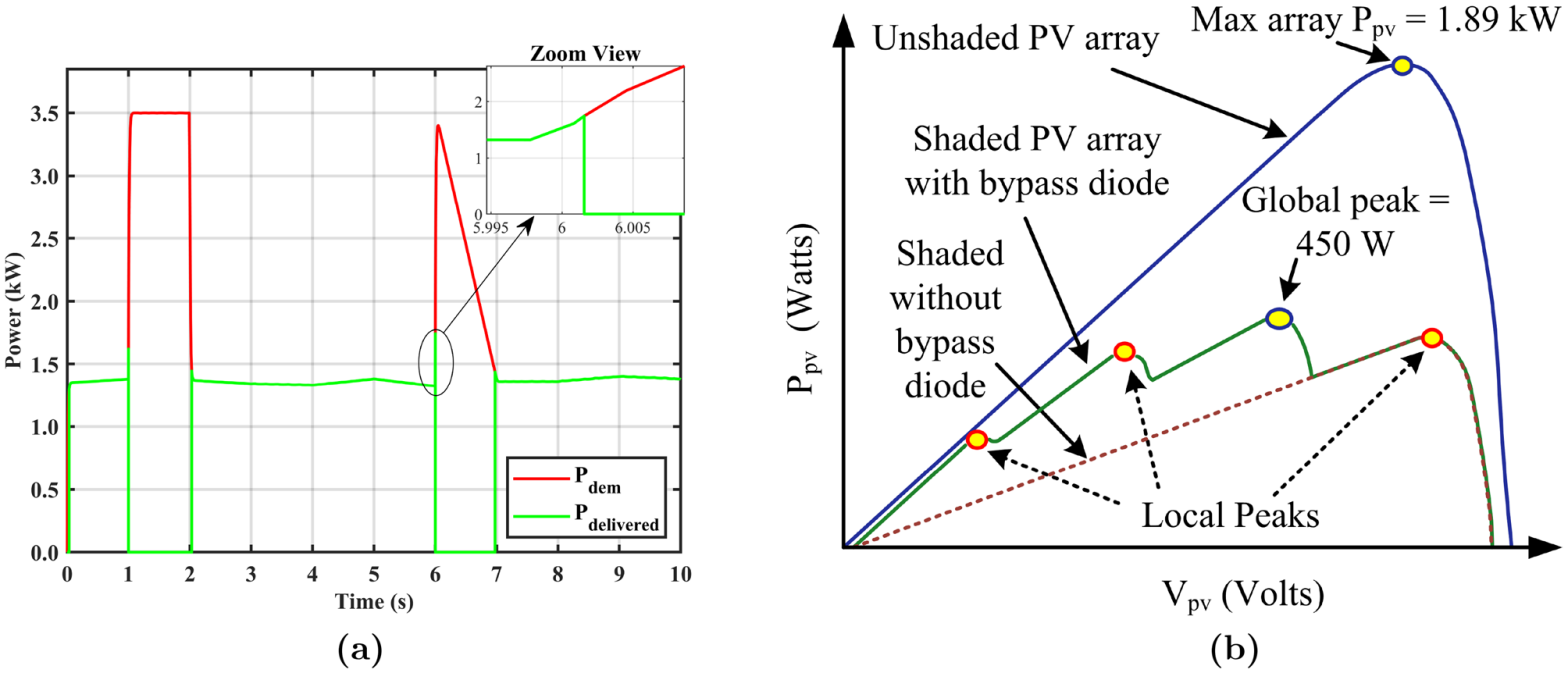
**(a)** PV without ESD driving PL, **(b)** PV under shaded and unshaded conditions.

The evaluation of the system is conducted without establishing any connection between an ESD and PV array. Figure 4a depicts that the PV system supplies a relatively consistent portion of the load within the prescribed limits. However, when a PL is directly applied at 1 s and 6 s, the system fails to sustain the load. It is due to the fact that the response of the array is slow, and the load pulse is greater than its capacity. Capacity can be increased by adding more PV modules, but it adds cost and weight to the system. Furthermore, if the system is designed solely on the basis of the PL requirements, any surplus energy generated during periods of reduced load will end up being wasted. In contrast, even if the capacity is expanded without cost and weight constraints, the ecological conditions persist. The system without ESD exhibits instability when subjected to low irradiance and shading conditions while the load is active. Therefore, it is strongly recommended to incorporate an ESD in the PV System to store excess energy during low-load scenarios and to serve as an energy source during low irradiance and PL demands.

### Variable PV with constant load

**Figure 5:**
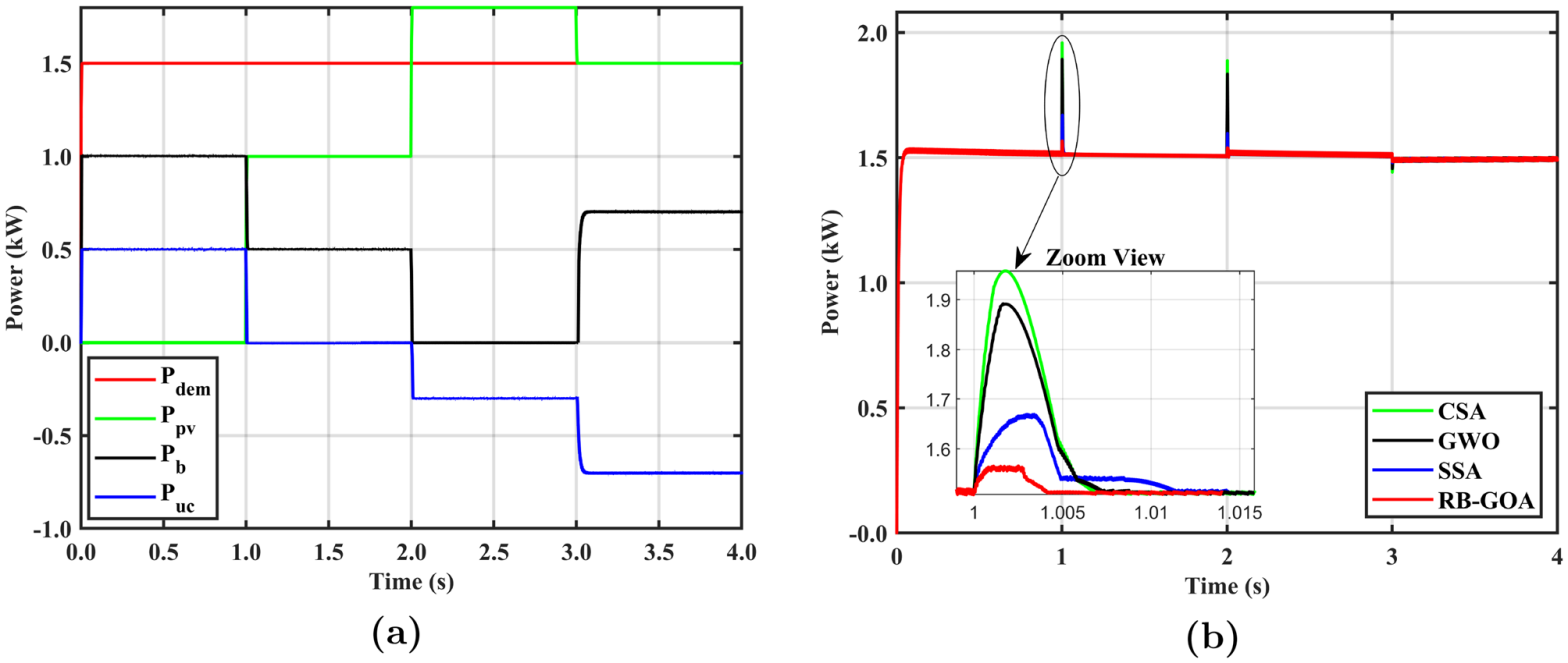
**(a)** $P_{dem}$ distribution among $P_{pv}, P_{b}, P_{uc}$ under variable PV with constant load case, **(b)** comparison of power delivered by SITs.

The proposed system is tested for a constant $P_{dem}$ of 1.5 kW with variable $P_{pv}$ as shown in Fig. 5a. In the initial 1 s, $P_{pv} = 0$ and $P_{dem} > P_b^{dlim}$ (the power demand exceeds the maximum power discharge limit of the BB); therefore, both the BB and the UC supply power to meet the load demand. The RB sets the values of $C_{pv}$ $\in$ [0,0], $C_b$ and $C_{uc}$ $\in$ [0,1] by enabling the BB2PL and UC2PL modes. The GOA takes this input from RB and optimizes the objective function by distributing power between BB and UC. It can be seen that the BB only provides power within its limits ($P_b^{dlim} = 1.0~kW$) while the remaining power is supplied by the UC ($P_{uc}$).

In the time interval 1 to 2 s, $P_{pv}$ is 1 kW but it is still insufficient to meet the $P_{dem}$. As the primary priority of PV system is to power the load, the PV2PL mode is activated. Furthermore, the BB2PL mode is also activated because the other condition $P_{dem} - P_{pv} < P_b^{dlim}$ is satisfied. The RB assigns values of $C_{pv}$ and $C_b$ $\in$ [0,1] and $C_{uc}$ $\in$ [0,0]. GOA distributes $P_{dem}$ between PV and BB. Consequently, all $P_{pv}$ is used to run the load and the remaining required power is delivered by BB ($P_b$). It is evident that $P_{pv} = 1~kW$, $P_b$ is reduced to 0.5 kW and the $P_{uc}$ becomes zero. Furthermore, between 2 and 3 s, when $P_{pv}$ reaches 1.8 kW, the condition $P_{dem} < P_{pv}$ is satisfied. This activates PV2PL as the first priority and initiates the PV2UC mode to store excess energy as $P_{dem} - P_{pv} < P_{uc}^{clim}$. The range of capacity factors is $C_{pv}$ $\in$ [0,1], $C_b$ $\in$ [0,0], and $C_{uc}$ $\in$ [-1,0]. The GOA assigns $P_{pv}$ to meet $P_{dem}$, $P_{uc}$ to charge the UC, and $P_b$ to zero. At the last interval, the load is solely powered by $P_{pv}$ in the PV2PL mode as $P_{dem} = P_{pv}$. In this situation, BB provides charging to the UC using the BB2UC mode. Table 4 presents the summary of system operation for this case.

**Table 4:** Summary of system operation for variable $P_{pv}$ with constant $P_{dem}$ case.

Time interval (s)	System operation
0–1	BB and UC supply power to load as $P_{pv} = 0$ and $P_{dem} > P_b^{dlim}$.
	Modes: BB2PL, UC2PL
1–2	PV and BB run the load as $P_{pv} = 1 kW < P{dem}$.
	Modes: PV2PL, BB2PL
2–3	PV runs the load alone as $P_{pv} = 1.8 kW > P_{dem}$ and excess energy is stored in UC.
	Modes: PV2PL, PV2UC
3–4	Load solely powered by $P_{pv}$ as $P_{dem} = P_{pv}$ and BB charges UC.
	Modes: PV2PL, BB2UC

Figure 5b depicts the power delivered by various SITs for the variable PV constant load case, where RB-GOA is compared with CSA, GWO and SSA. The results demonstrate the effectiveness of the proposed system in maximizing PV energy utilization and improving UC response during charging/discharging to alleviate stress on BB. For example, as $P_{pv}$ increases from 0 to 1 kW at 1 s, the BB reduces its discharging current and the UC shuts down its operation to fully use the available PV energy. The results demonstrate that the system experiences a substantial power surge during this mode transition at 1 s. In the case of CSA, the power surge of 0.45 kW lasting for 6.9 ms. For GWO, the surge is 0.39 kW lasting for 7.2 ms, while for SSA the surge is 0.18 kW lasting for 11.2 ms. In contrast, the RB-GOA exhibits fewer oscillations compared to other SITs with only a 0.05 kW power surge for 3.5 ms. The analysis shows that the proposed RB-GOA outperforms the other SITs by reducing power surge by 26% compared to CSA, 22% compared to GWO, and 8% compared to SSA. Furthermore, the oscillations in RB-GOA diminish twice as fast as in CSA and GWO, and more than three times as fast as in SSA. These results highlight the superiority of the RB-GOA over other SITs in minimizing power fluctuations during the mode transition and maintaining stable output across the load. Table 5 presents the comparison of the proposed RB-GOA with the other SITs for this case.

**Table 5:** Comparison of SITs for variable $P_{pv}$ with constant $P_{dem}$ case.

SIT	Attribute	
	Power surge during load transition (kW)	Surge die out time (ms)
CSA	0.45	6.9
Proposed (RB-GOA)	0.05	3.5
	RB-GOA reduces power surge by 9 times	RB-GOA is 2 times fast
GWO	0.39	7.2
Proposed (RB-GOA)	0.05	3.5
	RB-GOA reduces power surge by 8 times	RB-GOA is 2 times fast
SSA	0.18	11.2
Proposed (RB-GOA)	0.05	3.5
	RB-GOA reduces power surge by 3.6 times	RB-GOA is 3.2 times fast

### Variable PV with pulse load

**Figure 6:**
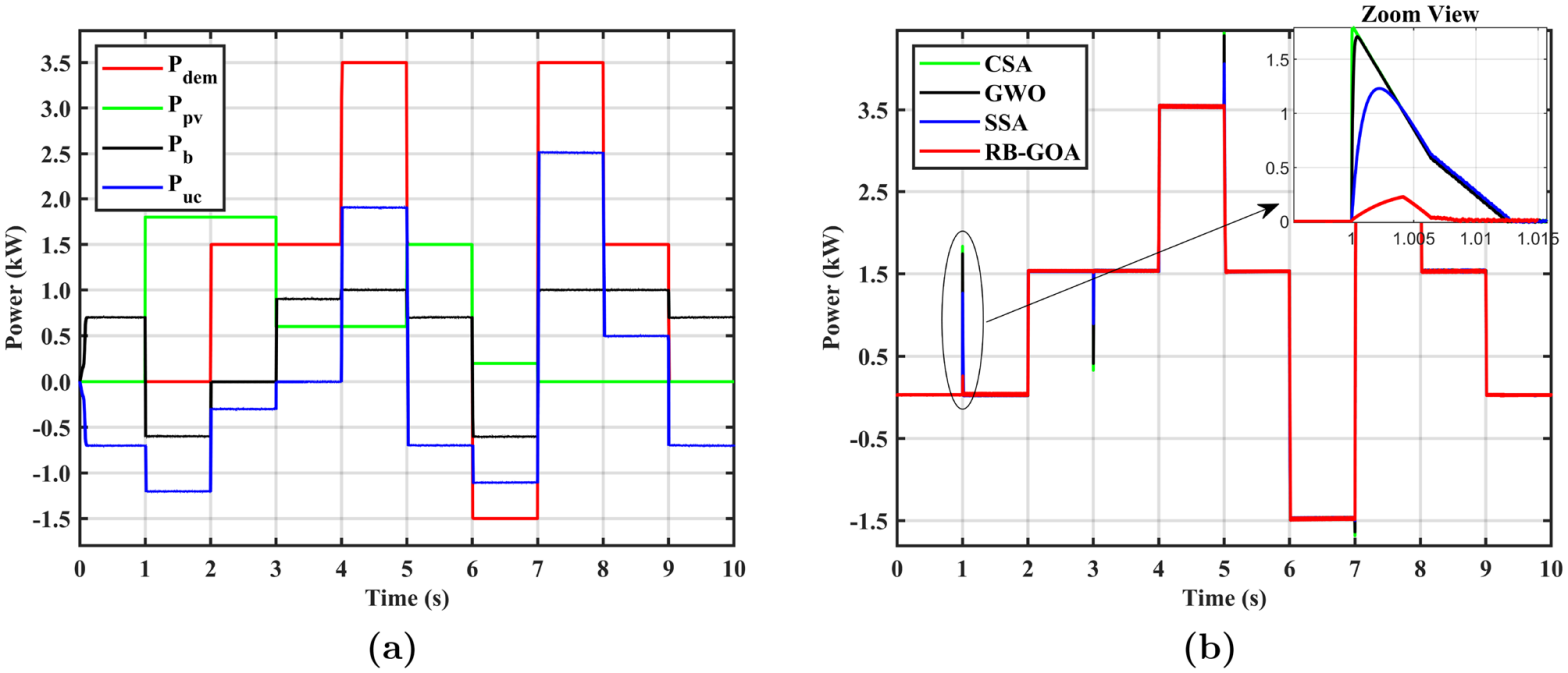
**(a)** $P_{dem}$ distribution among $P_{pv}, P_b, P_{uc}$ under variable PV with variable load conditions, **(b)** comparison of power delivered by SITs.

The proposed system is tested for a variable $P_{dem}$ of 0 to 3.5 kW with a variable $P_{pv}$ from 0 to maximum 1.89 kW as shown in Fig. 6a.

It can be seen that the system operates in an idle case ($P_{pv} = P_{dem} = 0$) during the initial 1 s. The RB establishes the values of the capacity factors $C_{pv}$ $\in$ [0,0], $C_b$ $\in$ [0,1], and $C_{uc}$ $\in$ [-1,0]. Furthermore, it activates the BB2UC mode, leading to the charging of the UC by the BB, which is managed by RB-GOA. In the time interval between 1 and 2 s, the $P_{pv}$ increases from 0 to a maximum of 1.89 kW and $P_{dem} = 0$. This activates the no-load case, in which the UC charging is given the highest priority activating the PV2UC mode. Moreover, $P_{pv}$ is more than the upper limit of the UC’s charging threshold ($P_{uc}^{clim}$) so, the PV2BB mode is also enabled to take maximum advantage of available $P_{pv}$. RB adjusts the values of $C_{pv}$ $\in$ [0,1], $C_b$ and $C_{uc}$ $\in$ [-1,0]. RB-GOA distributes the available $P_{pv}$ between the UC and BB, enabling the simultaneous charging of both ESDs using the entire output power of the PV source. Furthermore, when $P_{dem}$ increases to 1.5 kW at 2 s, the condition $P_{dem} < P_{pv}$ is satisfied which activates PV2PL in the first priority. Additionally, to store excess energy PV2UC mode is initiated as the available PV power is more than load demand and the condition $P_{pv} - P_{dem} < P_{uc}^{clim}$ is satisfied. The range of capacity factors is set to $C_{pv}$ $\in$ [0,1], $C_b$ $\in$ [0,0], and $C_{uc}$ $\in$ [-1,0]. The RB-GOA is responsible for dividing $P_{pv}$ between $P_{dem}$ to satisfy the priority load demand and $P_{uc}$ to charge the UC. There is no remaining power, so $P_{b}$ is zero. In the subsequent time intervals 3 to 4 s, $P_{pv}$ decreases to a point that condition $P_{dem} > P_{pv}$ becomes valid. This is the overload case in which $P_{pv}$ alone is insufficient to power the load. Therefore, RB-GOA takes all the available $P_{pv}$ and the remaining required power ($P_{dem} - P_{pv}$) from BB to meet the load demand. UC does not participate in the situation where the difference between $P_{dem}$ and $P_{pv}$) is less than the battery discharge limit ($P_{dem} - P_{pv} < P_b^{dlim}$). In this case, the two modes PV2PL and BB2PL are activated. An instant surge of $P_{dem}$ reaching 3.5 kW is observed at 4 s which is greater than the available ($P_{pv}$) and the discharge limit of BB ($P_b^{dlim}$). Thus, UC handles this surge in $P_{dem}$. In this situation, the three sources (PV, BB, and UC) are contributing to running the load, activating the PV2PL, BB2PL, and UC2PL modes with $C_{pv}$, $C_b$, and $C_{uc}$ $\in$ [0,1]. The RB-GOA distributes $P_{dem}$ among PV, BB, and UC based on their predefined limits. Moving on to the next interval (5 to 6 s), the equality case is considered in which $P_{dem} = P_{pv}$. In this case the load is solely run by $P_{pv}$ in PV2PL mode. At the same time, the BB charges the UC in the BB2UC mode. A regenerative case occurs in the time interval from 6 to 7 s in which $P_{dem} < 0$. During this interval, all $P_{dem}$ and $P_{pv}$ are used to charge the UC on priority. Since the regenerative energy and available PV energy ($|P_{dem}|+ P_{pv}$) exceeds the UC charging limit $P_{uc}^{clim}$, the BB also receives charging. In this case, the operating modes are PL2ESD and PV2UC, where $C_{pv}$ $\in$ [0,1], $C_b$ and $C_{uc}$ $\in$ [-1,0]. For the following time intervals (7 to 9 s), $P_{pv} = 0$ and $P_{dem}$ hold different values. The RB-GOA distributes the power share between UC and BB within their limits, as PV power is not available. In the final time interval, both $P_{dem}$ and $P_{pv}$ become 0. Therefore, the BB provides charging to the UC with the BB2UC mode. Table 6 presents the summary of system operation for this case.

**Table 6:** Summary of system operation for variable $P_{pv}$ with variable $P_{dem}$ case.

Time interval (s)	System operation
0–1	Idle case as $P_{pv} = P_{dem} = 0$. BB charges UC
	Modes: BB2UC
1–2	No-load case with $P_{pv}$ = 1.89 kW. PV charges UC and BB
	Modes: PV2UC, PV2BB
2–3	Underload case as $P_{dem} < P_{pv}$. PV runs the load and extra energy is stored UC
	Modes: PV2PL, PV2UC
3–4	Overload case as $P_{pv}$ is insufficient. BB provides the balance power as $P_{dem} - P_{pv} > P_b^{dlim}$
	Modes: PV2PL, BB2PL
4–5	Pulse power demand, UC handles surge in $P_{dem}$. PV, BB, UC contribute to load
	Modes: PV2PL, BB2PL, UC2PL
5–6	Equality case as $P_{dem}=P_{pv}$, load solely powered by $P_{pv}$ and UC gets charging from BB
	Modes: PV2PL, BB2UC
6–7	Regenerative case as $P_{dem} < 0$. UC and BB store excess energy ($|P_{dem}| + P_{pv} > P_{uc}^{clim}$)
	Modes: PL2ESD, PV2ESD
7–8	Pulse power demand with $P_{pv} = 0$. UC handles the power surge
	Modes: UC2PL, BB2PL
8–9	$P_{dem}$ is run by UC and BB as $P_{pv} =0$. RB-GOA distributes power share between ESD within their limits
	Modes: UC2PL, BB2PL
9–10	$P_{dem}$ and $P_{pv}$ become 0. UC gets charging from BB
	Modes: BB2UC

Figure 6b illustrates the power delivered by the different SITs for variable PV with variable load case. These results highlight the effectiveness of the proposed system in maximizing PV energy utilization and improving the UC response during charging and discharge, thus reducing the stress on the BB. As an example, as $P_{pv}$ increases from 0 to 1.89 kW, the UC receives a higher charging current, and the BB switches from discharging to charging mode to fully utilize the PV energy. The performance of the RB-GOA is compared with other SITs such as CSA, GWO, and SSA during this mode-change operation. The results demonstrate that the CSA exhibits a high surge in output power of 1.75 kW, GWO experiences a surge of 1.65 kW, and the SSA encounters a surge of 1.2 kW for 12 ms. In contrast, the RB-GOA exhibits fewer power oscillations with power surges of only 0.2 kW lasting for 5.5 ms. The analysis shows that the proposed RB-GOA outperforms the other SITs by significantly reducing power surges, achieving a 9-fold reduction compared to CSA and GWO, and a 6-fold reduction compared to SSA. Furthermore, the oscillations in RB-GOA diminish twice as fast as those in CSA, GWO, and SSA. These results highlight the superiority of the RB-GOA over other SITs in minimizing power surges during the mode transition and maintaining stable output across the load. Table 7 presents the comparison of the proposed RB-GOA with the other SITs for this case.

**Table 7:** Comparison of SITs for variable $P_{pv}$ with variable $P_{dem}$ case.

SIT	Attribute	
	Power surge during load transition (kW)	Surge die out time (ms)
CSA	1.8	12.8
Proposed (RB-GOA)	0.2	5.5
	RB-GOA reduces power surge by 9 times	RB-GOA is 2.4 times fast
GWO	1.65	12.3
Proposed (RB-GOA)	0.2	5.5
	RB-GOA reduces power surge by 8.2 times	RB-GOA is 2.2 times fast
SSA	1.22	12.7
Proposed (RB-GOA)	0.2	5.5
	RB-GOA reduces power surge by 6 times	RB-GOA is 2.3 times fast

**Figure 7:**
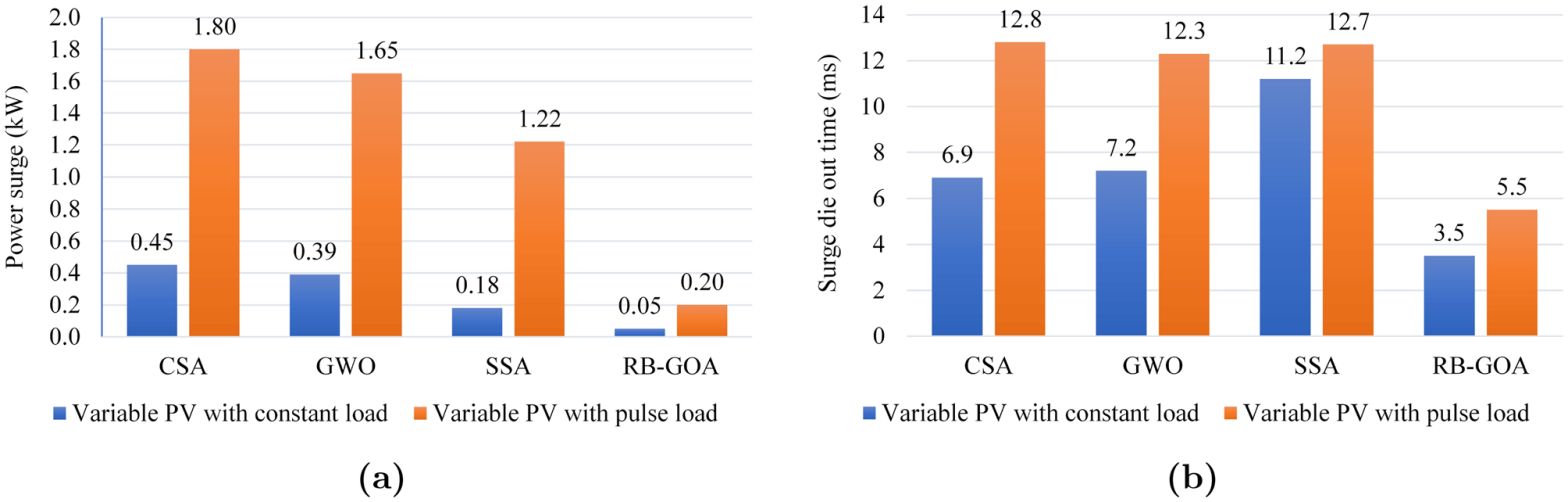
**(a)** Power surge during load transition, **(b)** surge die-out time.

Figure 7 presents the graphical representation of the results obtained for variable PV with constant load and variable PV with pulse load cases. Figure 7a shows the comparison of power surges (kW) appeared in optimization processes during load transition for both cases using SITs. Furthermore, the surge die-out time (ms) for the same set of optimization techniques during load transitions is presented in Fig. 7b. The graph provides insight into the time it takes for the power surge to dissipate for the specified load transition scenarios. These figures offer a visual overview of the comparative performance of optimization techniques in managing power surges and surge die-out times during load transitions, discussed in the corresponding sections.

### PV array with MPPT

One of the challenges with PV-based hybrid systems is the lack of consideration for MPP tracking. In this study, MPP is tracked using GOA and its performance is compared with CSA, GWO, and SSA. Figure 4b illustrates two conditions to verify the performance of PVS under uniform irradiance (non-shaded condition) and non-uniform condition (PSC). Under uniform conditions, the maximum power of 1.89 kW can be achieved by connecting six PV modules (SPR-315E-WHT-D) in the matrix configuration using standard test conditions (STC) which are G = 1000 $\hbox {W}\,{\hbox {m}}^{-2}$ and $T=25^{\circ }$C. In the second case, four peaks are observed under PSC with a global peak (GP) at 450 W among the other three local peaks.

**Figure 8:**
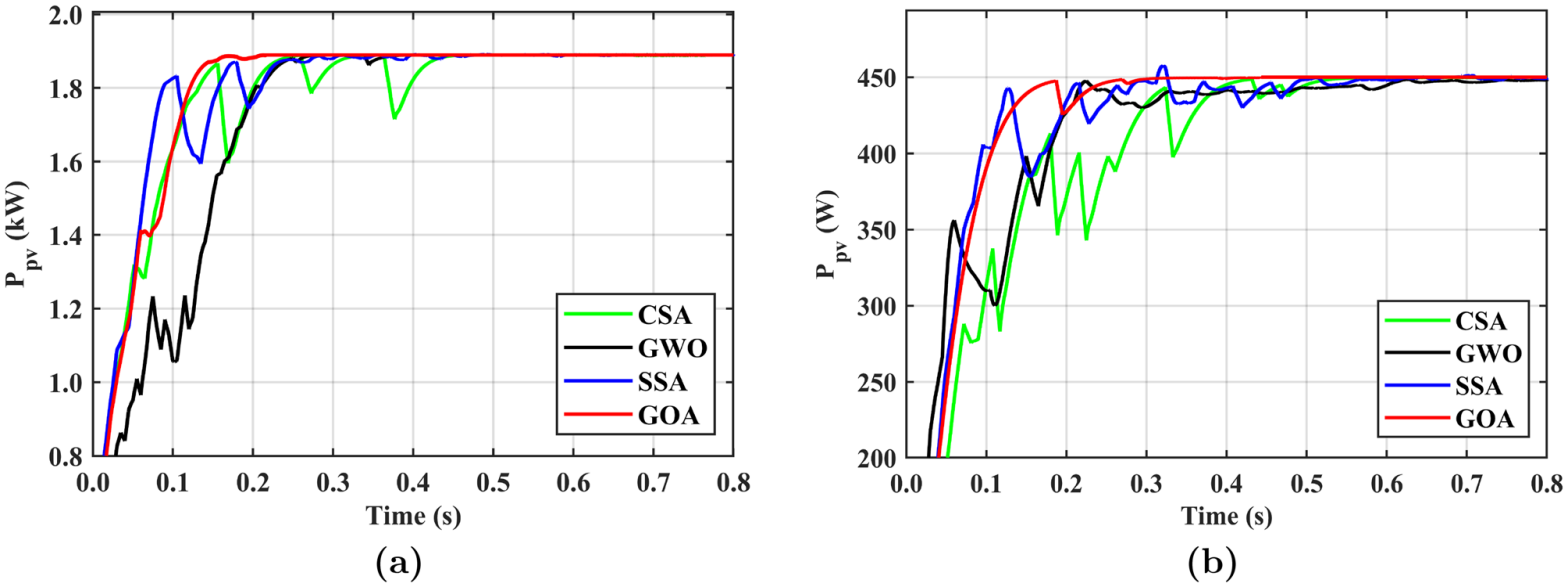
PV with MPPT **(a)** under uniform irradiance, **(b)** under PSCs.

In the first case depicted in Fig. 8a, the tracking time for CSA exceeds 0.15 s, and the settling time is 0.47 s with more oscillation compared to the other SITs employed in this study. The GWO algorithm achieves GP tracking at 0.28 sand settles the output at 0.35 s, with initially higher oscillations that gradually decrease. Similarly, the SSA exhibits tracking and settling times of 0.18 s and 0.28 s, respectively, with fewer oscillations compared to CSA and GWO. On the other hand, the GOA achieves tracking in just 0.14 s and settles the output in 0.15 s without oscillations. It is evident that GOA outperforms the other SITs, demonstrating lower tracking time, settling time, and oscillations. In the case of a PSC with four peaks, as shown in Fig. 8b, the CSA is slowest among the other three SITs, taking 0.44 s for tracking and 0.54 s to settle the PV output. It exhibits higher oscillations, making it a less suitable choice for applications that demand fast and smooth output. On the other hand, the GWO achieves faster tracking speed taking only 0.23 s with a settling time 0.67 s is longer than the CSA with lower oscillations. The SSA demonstrates shorter tracking and settling times of 0.22 s and 0.29 s respectively than those of GWO and CSA. GOA is the fastest among the other three SITs, taking only 0.17 s for tracking and 0.27 s for settling time with minimal oscillations in the output curve. It is evident that GOA is approximately 61% faster than CSA, 35% faster than GWO, and 29.41% faster than SSA in uniform irradiance and partial shading conditions. Table 8 summarises the MPPT results and SITs comparison.

**Table 8:** MPPT results comparison of SITs.

SIT	Uniform irradiance			PSC with 4 peaks		
	${t}_{track}$ (s)	${t}_{setl}$ (s)	*Osc*	${t}_{track}$ (s)	${t}_{setl}$ (s)	*Osc*
CSA	0.16	0.47	H	0.44	0.54	H
GWO	0.28	0.35	M	0.23	0.67	M
SSA	0.18	0.28	L	0.22	0.29	M
Proposed	0.14	0.15	VL	0.17	0.27	VL

${t}_{track}$: Average tracking time, ${t}_{setl}$: Average settling time.

*Osc*: Oscillations, VL: Very low, L: Low, M: Medium, H: High.

The proposed system holds significant importance in the energy sector, particularly regarding PV energy integration. This research has practical implications and addresses crucial aspects:

- *Reducing power surges and oscillations:* EMS helps to maintain the stability of the energy system by reducing surges and mitigating oscillations that can damage equipment and disrupt power supply.
- *Improved MPPT speed: *Achieving maximum energy from PV arrays requires a faster MPPT speed. EMS optimizes the process, ensuring energy generation closely follows solar irradiance changes, maximizing PV system efficiency.
- * Efficient integration of PV energy with ESDs:* Efficient integration of PV energy with BB and UCs is a significant step toward ensuring a stable and sustainable energy supply. EMS regulates energy flow among these components, enhancing the overall efficiency of the system.
- *Adaptation to dynamic PV systems:* The inclusion of both long-term energy management and short-term power management in EMS allows it to adapt dynamically to the ever-changing nature of PV systems and loads. This adaptability ensures a consistent energy supply, particularly in variable weather conditions.
- * Optimized real-time power sharing:* The proposed approach optimizes real-time power sharing among PV, BB and UCs, promoting efficient energy utilization and minimizing waste.
- *Fresh perspective on SITs in BB-UC hybrid systems:* This work provides a new perspective on the application of SIT in solar-powered BB-UC hybrid systems, offering advanced solutions to energy management challenges in such setups.

The physical significance of this research lies in its practical applicability to real-world energy systems. It incorporates a control mechanism that introduces damping in the system during load transitions, similar to shock absorbers in a vehicle. This physical damping reduces the rate of change of power, effectively reducing the surges and oscillations in the system. Additionally, UCs act as an energy buffer, rapidly releasing and absorbing energy to maintain system stability during a load transition. This inertia in energy transfer physically reduces the rapid changes in power. The proposed technique reduces power surges by providing UCs for the surge current to flow, allowing it to dissipate quickly and safely into the system. It can be used in a variety of applications, including power grids, industrial motors, wind turbines, and PV systems.

## Conclusion

The proposed EMS based on RB-GOA to support pulse load exhibits outstanding performance compared to CSA, GWO, and SSA in terms of reducing power surges during load transition, mitigating oscillations, and achieving faster MPPT speed. It reduces power surges by 26% compared to CSA, 22% compared to GWO, and 8% compared to SSA during load transitions in the variable PV constant load case. It also mitigates oscillations twice as fast as CSA and GWO, and more than three times as fast as SSA. In the variable PV variable load case, it shows a significant reduction in power surges by 9 times compared to CSA and GWO, and by 6 times compared to SSA. Additionally, it achieves approximately 61% faster MPPT speed than CSA, 35% faster than GWO, and 29% faster than SSA, regardless of weather conditions. The work efficiently addresses challenges of high response times, output fluctuations, and integration of PV with ESDs. It efficiently regulates energy flow among PV systems, BB, and UCs using an EMS. The EMS includes both long-term energy management and short-term power management to adapt to the dynamic nature of PV systems. Furthermore, RB-GOA optimizes real-time power sharing among energy components and provides a fresh perspective on SITs in BB-UC hybrid systems. Moreover, an optimized MPPT scheme maximizes the energy harvesting from the PV array and mitigates the effect of varying solar irradiance. This multifaceted contribution offers valuable information on improving the performance and reliability of solar-powered BB-UC HESS under dynamic real-world conditions.

## Data Availability

The datasets used and/or analysed during the current study available from the corresponding author on reasonable request.
